# Advanced Thyroid Cancer Patients in Phase I Clinical Trials: Outcome Assessment and Literature Review

**DOI:** 10.14740/wjon768w

**Published:** 2014-03-11

**Authors:** Laeeq Malik, Alex Mejia

**Affiliations:** a Institute for Drug Development (IDD), Cancer Therapy and Research Center (CTRC), University of Texas Health Science Center, San Antonio, TX, USA

**Keywords:** Thyroid, Cancer, Phase I, Trials

## Abstract

**Background:**

To describe the basic demographics, analyze the response and survival experience of advanced thyroid cancer subjects treated in Phase I clinical trials. We also reviewed early Phase studies using new targeted agents in thyroid cancer.

**Methods:**

We conducted a retrospective observational study in 32 advanced thyroid cancer patients who participated in 21 Phase I trials at our center between 2002 and 2012. Descriptive statistics and Kaplan Meier analyses were used to examine clinical outcomes and survival.

**Results:**

The median age of subjects was 57.5 years (range 21-81) at the time of study enrollment; more men (53.1%) than women were enrolled. A large number of study participants were Hispanic Americans. Nearly half (46.8%) of patients had ECOG performance status of zero, 53.1% were ECOG one and above. The most common histological subtypes were papillary (37.5%), medullary (28.1%), follicular (18.7) and anaplastic (15.6%). More than half of patients had ≥ 2 metastatic sites (62.6%). Of the 30 evaluable patients for tumor response, 2 confirmed partial responses (6.6%) were observed, whereas 17 patients had stable disease (SD) as best response. Among patients with stable disease, 10 patients (33.3%) achieved SD lasting ≥ 6 months. The median overall survival from the time of enrollment in a Phase I trial was 16.1 months. No treatment related death was observed among these patients treated with an investigational agent.

**Conclusion:**

Advanced thyroid cancer patients with no standard treatment options could participate in early Phase clinical trials of novel agents.

## Introduction

Thyroid cancer is the most common malignancy of the endocrine system and affects women more often than men [1]. Around 60,220 new cases of thyroid cancer are expected to be diagnosed in the United States in 2013 [2]. It is a heterogeneous disease that is classified into differentiated (DTC), anaplastic (ATC) and medullary thyroid cancers (MTC). DTC including papillary (PTC), follicular (FTC) and Hurthle cell carcinomas (HTC) is the most common histological subtype (85%) [1]. Total thyroidectomy or lobectomy is the surgical therapy of choice with similar survival results for both procedures [3]. Radioactive Iodine (RAI) is used in patients with DTC as an adjuvant therapy in post-thyroidectomy setting or when microscopic residual cancer is suspected. RAI also causes thyroid cell death by emitting beta radiation in patients with recurrent and metastatic disease. The overall prognosis in patients with DTC is very good; however, some patients with high risk features develop local or distant recurrence. Recurrent disease is treated by surgical resection, RAI, external radiotherapy, thyroid hormone therapy, chemotherapy or more recently targeted therapy depending upon the site and extent of disease.

Patients with RAI resistant metastatic disease have poor overall outcome with a 10-year survival rate less than 15% [4]. Systemic chemotherapy for advanced thyroid cancer has limited effectiveness with some suggestion of efficacy in anaplastic carcinoma [5, 6]. Clinical trials with novel agents are recommended for patients with advanced thyroid cancer and distant metastasis that is unresponsive or not amenable to the above mentioned treatments [7, 8]. Currently there is limited published literature on the treatment outcomes of patients with advanced thyroid cancer treated in Phase I clinical trials. We retrospectively analyzed data from 32 consecutive patients with advanced thyroid cancer who received treatment with novel agents in Phase I clinical trials at our cancer center between 2002 and 2012.

## Materials and Methods

### Patients and data acquisition

The Cancer Therapy and Research Center (CTRC) is a tertiary care cancer center in San Antonio, Texas. It has a well-established Institute for Drug Development with a particular focus on Phase I clinical trials since the early 1990s. The University of Texas Health Science Center at San Antonio (UTHSCSA) has an informatics data exchange and acquisition program, which serves as a primary research data system. All thyroid cancer patients participating in Phase I clinical trials at CTRC from January 2002 to December 2012 were identified through this system. All patients completed an informed consent process prior to enrollment onto a trial and all trials were approved by the UTHSCSA Institutional Review Board.

All patients with advanced thyroid cancer of any histological subtype, who were successfully enrolled in a Phase I study, were included in this review. Patients who failed screening process were excluded from this analysis. Patient’s electronic medical records from the initial clinic visit to the time of last visit were reviewed. We extracted demographic data (gender, age); medical information (disease site, tumor histology, date of diagnosis of initial and metastatic disease, number and nature of prior treatments, performance status); details of Phase I trial (nature of investigational agent, date of consent, date and reason for removal from study); information on clinical outcome, subsequent treatment; and laboratory data from physicians’ clinical notes that were dictated at the time of clinic visit. All the data were entered into a password-protected database.

### Outcomes

Progression free survival (PFS) was measured from study enrollment to the date when the patient was removed from study for progression or death. Patients who were still continuing on treatment at the time of last follow-up were censored on that date. Survival was measured from the date of enrollment in study until death from any cause. Survival was also estimated from the date of diagnosis of recurrent/metastatic disease until death. Patients who were still alive at the time of last follow-up were censored on that date.

### Statistical analysis

Descriptive statistics were used to describe patients’ demographic and treatment characteristics by outcome (partial response, stable disease, progressive disease). Survival was plotted using Kaplan Meier method. A P-value < 0.05 was considered statistically significant in all analyses. Statistical analysis was performed using IBM SPSS Statistics 20 software (Armonk, NY, USA).

## Results

### Pre-enrollment characteristics

The median age of subjects was 57.5 years (range 21-81) at the time of study enrollment; more men than women were enrolled (53.1% versus 46.8%). A large number (43.7%) of study participants were Hispanic Americans. Nearly half (46.8%) of patients had ECOG performance status of zero, 53.1% were ECOG one or above. The most common histological subtypes were papillary (37.5%), medullary (28.1%), follicular (18.7) and anaplastic (15.6%). More than half (62.6%) of patients had ≥ 2 metastatic sites; 68.7% had lung, 37.5% had bone and 21.8% had liver involvement. All of the patients had a history of prior thyroidectomy. A quarter (25%) of subjects had received ≥ 2 prior anticancer therapies for the metastatic disease, including a combination of chemotherapy and biological therapy. Other baseline characteristics are summarized in Table 1.

**Table 1:** Baseline Characteristics

Age	
Median (range)	56 (21-81)
Sex (no, %)	
Female	15 (46.8)
Male	17 (53.1)
Ethnicity (no, %)	
White	11 (34.3)
Hispanic	14 (43.7)
Other	7 (21.8)
ECOG performance status (no, %)	
0	15 (46.8)
≥1	17 (53.1)
Tumor histology (no, %)	
Papillary	12 (37.5)
Follicular	6 (18.7)
Medullary	9 (28.1)
Anaplastic	5 (15.6)
No of metastatic sites (no, %)	
1	12 (37.5)
2	10 (31.3)
≥3	10 (31.3)
Site of metastases (no, %)	
Lung	22 (68.7)
Bone	12 (37.5)
Lymph node	10 (31.2)
Liver	7 (21.8)
Mediastinum	7 (21.8)
No of prior systemic treatments (no, %)	
0	6 (18.7)
1	18 (56.2)
≥2	8 (25)
Type of prior treatment (no, %)	
Thyroidectomy	32 (100)
Iodine-131	21 (65.6)
Chemotherapy	19 (59.3)
Biological	10 (31.2)
Type of Phase I trial (no, %)	
Single biological agent	24 (75.0)
Combination biological therapy	6 (18.7)
Combination of Chemotherapy and biological agent	2 (6.2)
Reason to come off study (no, %)	
Progression	20 (62.5)
Toxicity	5 (15.6)
Patient preference/Other	7 (21.8)
Treatment on progression (no, %)	
Another trial	13 (40.6)
Off trial treatment	8 (25.0)
No treatment/supportive care	7 ( 21.8)
Unknown	4 (12.5)

### Treatment and trials

In total, the 32 patients included in this analysis were treated on 21 Phase I trials; 30 were evaluable for a treatment response. Twenty-four patients were treated with a single targeted agent, six patients were treated with a two-drug targeted therapy combination, and two patients were treated with chemotherapy. Among the 21 Phase I trials, 16 investigated single agents whilst five evaluated different combinations. Five trials investigated an antibody or small molecule targeting vascular endothelial growth factor receptor (VEGFR) or/and multiple receptortyrosine kinases; four trials involved agents targeting epidermal growth factor receptors (EGFR); four investigated agents targeting histone deacetylation (HDAC); two used agents targeting mammalian target of rapamycin (mTOR); two used chemotherapy agents (carboplatin and paclitaxol); and four involved agents with miscellaneous targets (NF-kappa B, polo-like protein kinase, clusterin, insulin growth factor receptor).

### Response

Best radiological response was assessed by serial CT or MRI scan using Response Evaluation Criteria in Solid Tumors (RECIST) version 1.0 or 1.1 [9, 10]. Imaging was performed approximately every two or three cycles depending upon the individual study protocol. In patients with measurable disease, the response was classified as complete (CR), partial (PR), stable (SD) or progressive disease (PD).

Of the 30 patients evaluable for tumor response, two confirmed PRs (6.6%) were observed, whereas 17 patients had SD as best response. Among patients with stable disease, 10 patients (33.3%) achieved SD lasting ≥ 6 months. There were three patients (10%) who received treatment for ≥ 12 months. Thirteen patients (43.3%) were found to have clinical/radiological PD before or at the time of first tumor assessment. The total number of patients with PR or SD lasting more than 6 months was 12 (40%).

The two patients with PR were Hispanic males with a median age of 43 years. One patient had PTC with pulmonary metastases that had previously progressed on tyrosine kinase inhibitor after 6 weeks. He received a HDAC inhibitor for a total of 49 months before meeting the criteria for disease progression. The second patient had MTC with liver metastases that had previously progressed on chemotherapy within 8 weeks. He received sorafenib in combination with temsirolimus for 18 months before progression of liver disease.

### Outcome characteristics and prognostic factors

**Figure 1:**
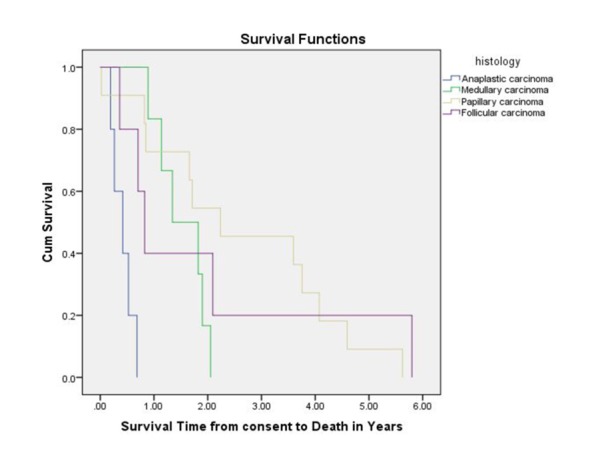
Overall survival measured from Phase I study enrollment until death.

Among 32 patients, there were 27 deaths. Three patients were still alive and survival information was missing for two patients. The median overall survival (OS) from the time of enrollment in a Phase I trial was 16.1 months (Fig. 1). With regard to the different histological subtypes, the median OS measured 5.0 months for ATC, 9.9 months for FTC, 16.1 months for MTC and 26.8 months for PTC. The median OS from the time of diagnosis of metastatic disease was 55.8 months. Thirteen subjects survived for more than 24 months after enrolling in a Phase I study. Patients with a PR had higher OS as compared to patients with SD or PD. The median PFS measured 11.6 weeks for ATC, 10.8 weeks for PTC, 14.4 weeks for FTC and 31.6 weeks for MTC. The duration of response (PR or SD) to the Phase I agent was longer when compared to the duration on prior chemotherapy for most patients (Table 2).

**Table 2:** Patients With Partial Responses or Stable Disease

Phase I Drug target	Tumor type	Best response	Duration of response in Phase I trial (weeks)	Duration of response on prior therapy (weeks)
HDAC	PTC	PR	196	6
VEGFR	MTC	PR	72	8
EGFR	ATC	SD	9	NA
HDAC	PTC	SD	24	20
HDAC	MTC	SD	35	22
mTOR	MTC	SD	33	NA
EGFR/HER2	MTC	SD	25	14
EGFR/HER2	MTC	SD	14	16
EGFR/HER2	MTC	SD	30	18
mTOR	FTC	SD	20	15
EGFR	ATC	SD	17	4
EGFR	PTC	SD	16	9
VEGFR	PTC	SD	42	NA
VEGFR	FTC	SD	48	6
Microtubule	MTC	SD	12	10
VEGF	PTC	SD	9	20
IGF-IR	FTC	SD	29	22
IGF-IR	MTC	SD	36	16
EGFR	MTC	SD	24	14

Abbreviations: MTD: Maximally tolerated dose; PTC: Papillary thyroid cancer; MTC: Medullary thyroid cancer; ATC: Anaplastic thyroid cancer; FTC: Follicular thyroid cancer; PR: Partial response; SD: Stable disease; HDAC: Histone deacetylase; VEGFR: Vascular endothelial growth factor receptor; EGFR: Epidermal growth factor receptor; HER2: Human epidermal growth factor receptor 2; mTOR: Mammalian target of rapamycin; IGF-IR: Insulin-like growth factor receptor; NA: Not available)

### Safety and toxicity

No treatment related death was observed among these patients while being treated with an investigational agent. Treatment at the assigned dose level was well managed, dose reduction was only required for 15.6% patients. Five patients came off study for toxicity reasons. No anaphylactic reaction to investigational agent was observed. The most common grade 3 and greater toxic effects were fatigue (31.2%), hand foot syndrome (28%), diarrhea (14.3%), hypertension (12%), neutropenia (9.5%), thrombocytopenia (8.5%) and mucositis (5.2%).

## Discussion

Until more recently, there has been no effective treatment for RAI refractory advanced thyroid cancer. With the ongoing development of novel targeted therapies, there has been a growing interest in the inclusion of thyroid cancer patients in Phase I studies. Our data show that the PFS for thyroid cancer patients in Phase I studies is longer compared to the PFS on their prior chemotherapy. In the chemotherapy era, PFS for patients with metastatic DTC was estimated to be 2 months and median overall survival time was 8 months [11]. Thus, our survival results may represent a considerable improvement in outcome for these patients. It is worth noting that none of these 21 agents have yet received regulatory approval for a use in this indication.

The most common sites of metastatic thyroid cancer are the lung and bone. It is paramount to diagnose recurrent disease at the earliest opportunity in order to offer curative surgery or RAI. The management of metastases that do not take up RAI or metastatic MTC (relatively radio-resistant) remains challenging [12]. Systemic chemotherapy for metastatic thyroid cancer has a low response rate compared to other malignancies and considerable toxicity [13]. Novel treatment approaches that can provide clinically important benefits for appropriately chosen subsets of patients with advanced thyroid cancer are needed. The angiogenic pathway represents an important therapeutic target in DTC. In the recent years, over-activation of receptor tyrosine kinases (RTK), downstream signaling molecules, and inhibition of apoptosis have all been demonstrated to occur in thyroid cancer. With the identification of mutations involving RTK, RAS, RET and BRAF genes, there has been increasing focus on the rapid development of molecularly targeted agents blocking these specific pathways [14, 15].

In addition to the review of the Phase I thyroid cancer patients treated at CTRC, we performed a literature review to explore the successes, limitations and future challenges in treating advanced thyroid cancer, paying particular attention to the development of the targeted therapy in this area of unmet need. We aimed to identify prospective therapeutic clinical trials undertaken in advanced thyroid cancers and published in peer-reviewed journals. We used general search strategies to identify articles, primarily in PubMed, including the search terms “thyroid cancer trials”, “targeted therapy in thyroid cancer” and “new drugs in thyroid cancer”. Articles were individually reviewed. These data were not combined or subjected to meta-analysis. We hereby summarize the results of important early Phase studies of new agents in thyroid cancer.

Recent efforts have mainly focused on targeting vascular endothelial growth factor (VEGF), HDAC, PI3K-Akt-mTOR and mitogen-activated protein kinase (MAPK) pathways. Tyrosine kinase inhibitors (TKI) have opened a new era in the RAI refractory thyroid cancer. Vandetanib and cabozantinib-s-malate have recently been approved by the US Food and Drug Administration (FDA) as treatment for MTC. No comparative clinical trials of TKIs have yet been undertaken in this patient population. Vandetanib is a tyrosine kinase inhibitor with activity against RET, VEGFR-2 and EGFR. Based on the clinical and pharmacokinetic results in the initial Phase I study, an oral dosing of 300 mg/day was further evaluated in a Phase III trial [16]. Vandetanib showed significant activity with a response rate (RR) of 45% and prolongation of PFS in patients with the hereditary or the sporadic form of MTC as compared to placebo [17]. Furthermore, among the patients randomized to the placebo arm and after being switched over to open-label vandetanib, 12 patients had an objective tumor response. Cabozantinib-s-malate is also an oral inhibitor of MET, VEGFR2 and RET kinase. Of 35 patients with MTC included in a Phase I trial, 17 experienced a PR [18]. Subsequently a Phase III trial by Elisei et al reported a PR rate of 28% and median PFS of 11.2 months in advanced MTC [19]. All patient subgroups demonstrated prolongation of PFS, including those with prior TKI treatment. Common adverse events included diarrhea, palmar-plantar erythrodysesthesia, decreased appetite, nausea and fatigue.

Other non-approved TKIs currently being evaluated in early phase clinical trials include sorafenib tosylate, pazopanib hydrochloride, motesanib diphosphate, sunitinib malate, lenvatinibmesylate and axitinib. At present there is no standard therapy for patients with progressive DTC. Currently the FDA is giving priority review to a New Drug Application (NDA) for sorafenib tosylate as treatment for patients with advanced thyroid cancer. Sorafenib tosylate is an oral, multitargeted TKI against VEGFR, RET and BRAF with a response rate of 7-25% in nonrandomized studies [20-22]. In a recently completed Phase III trial of 417 patients with metastatic DTC, PR was seen in 12.2% patients and PFS of 10.8 months [23]. Median overall survival has not been reached; however, a benefit in survival is unlikely to emerge primarily because the majority of the patients in the placebo arm were crossed over to sorafenib treatment.

Pazopanib hydrochloride is another oral angiogenesis inhibitor targeting VEGFR, platelet derived growth factor receptor (PDGFR) and c-Kit. In the initial dose escalation Phase I study, prolonged stable disease lasting > 6 months was observed in one thyroid cancer patient [24]. Since then responses have been observed in all thyroid histological subtypes in a Phase II trial of RAI refractory thyroid cancer [25]. Motesanib diphosphate is an oral inhibitor of VEGFR, PDGFR and c-Kit. In a Phase I study, daily treatment with 125 mg of motesanib diphosphate resulted in antitumor activity in patients with advanced solid cancers, including five patients with thyroid cancer [26]. An efficacy signal was also observed in a Phase II study in which 13 patients with metastatic DTC had a PR [27]. Other anti-angiogenic inhibitors have also shown encouraging efficacy signal in clinical trials [28, 29].

Alteration in HDAC activity has been reported in several tumors, including thyroid cancer. In preclinical studies, HDAC inhibitors induced apoptosis in several cancer cell lines [30, 31]. In an initial Phase I trial of vorinostat, six patients with thyroid cancer maintained stable disease as best response for a median of 27 months [32]. These promising results in thyroid cancer were not reproduced in a later study investigating a schedule of 200 mg twice daily for 2 weeks, followed by 1 week off (3 weeks cycle) [33]. In order to improve efficacy, future research could focus on a continuous dosing schedule or combination therapy with doxorubicin and paclitaxel to exploit a possible synergy [31].

Multiple critical cellular functions are controlled by the PI3K-Akt-mTOR pathway. Several targets of mTOR have been found to be deregulated in thyroid cancer, making it an appropriate target for thyroid cancer research. Inhibition of this target can induce a significant dose-dependent growth inhibition in thyroid cancer [34]. Although everolimus has modest clinical activity as a single agent, some patients can obtain durable clinical benefit [35]. Combination therapy with everolimus and MEK inhibitor could further enhance antitumor activity by overcoming the adaptive resistance of cancer cells and blocking alternate signaling pathway.

The BRAF V600E mutation is found in more than 50% of all thyroid malignancies (primarily PTC) [36, 37]. Following Ras activation, Raf (downstream effector) phosphorylates MAPK, thus initiating a cascade of events resulting in cell proliferation. Following encouraging results with vemurafenib in the initial study, patients with progressive RAI-refractory BRAF V600E-mutant PTC in a Phase II trial demonstrated a PR rate of 35% and median PFS of 15.6 months [38, 39]. The benefit was higher in the group without prior VEGF therapy. Another BRAF inhibitor, dabrafenib has also shown promising clinical activity as a single agent in BRAF-mutant metastatic PTC [40]. A randomized phase II trial comparing dabrafenib with dabrafenib plus the MEK inhibitor is ongoing in this cancer subtype. Some early clinical data is also available for single agent MEK inhibitors such as selumetinib [41].

These recently identified therapeutic molecular targets and markers, lead us to an exciting modern era of molecular medicine for thyroid cancer. Development of drugs targeted to particular subgroups represents a major way forward in cancer therapy over the last decade. Just as we have learned from the experience in developing chemotherapy regimens, combining targeted therapies may further improve the outcomes of thyroid cancer patients. Hence enrollment of patients with thyroid cancer into Phase I and II studies should be encouraged in order to move into the forefront of management of this disease.
